# Association of Health Literacy Among Nulliparous Individuals and Maternal and Neonatal Outcomes

**DOI:** 10.1001/jamanetworkopen.2021.22576

**Published:** 2021-09-01

**Authors:** Lynn M. Yee, Robert Silver, David M. Haas, Samuel Parry, Brian M. Mercer, Deborah A. Wing, Uma Reddy, George R. Saade, Hyagriv Simhan, William A. Grobman

**Affiliations:** 1 Division of Maternal-Fetal Medicine, Department of Obstetrics and Gynecology, Northwestern University Feinberg School of Medicine, Chicago, Illinois; 2 Division of Maternal-Fetal Medicine, Department of Obstetrics and Gynecology, The University of Utah, Salt Lake City; 3 Department of Obstetrics and Gynecology, Indiana University School of Medicine, Indianapolis; 4 Division of Maternal-Fetal Medicine, Department of Obstetrics and Gynecology, Perelman School of Medicine, University of Pennsylvania, Philadelphia; 5 Department of Obstetrics and Gynecology, The MetroHealth System, Case Western Reserve University, Cleveland, Ohio; 6 Division of Maternal-Fetal Medicine, Department of Obstetrics and Gynecology, University of California, Irvine; 7 Division of Maternal-Fetal Medicine, Department of Obstetrics, Gynecology, and Reproductive Science, Yale University, New Haven, Connecticut; 8 Division of Maternal-Fetal Medicine, Department of Obstetrics and Gynecology, University of Texas Medical Branch, Galveston; 9 Department of Obstetrics, Gynecology, and Reproductive Science, University of Pittsburgh School of Medicine, Pittsburgh, Pennsylvania

## Abstract

**Importance:**

Health literacy is considered an important social determinant of health that may underlie many health disparities, but it is unclear whether inadequate health literacy among pregnant individuals is associated with adverse maternal and neonatal outcomes.

**Objective:**

To assess the association between maternal health literacy and maternal and neonatal outcomes among nulliparous individuals.

**Design, Setting, and Participants:**

This was a secondary analysis of a large, multicenter cohort study of 10 038 nulliparous individuals in the US (2010-2013). Participants underwent 3 antenatal study visits and had detailed maternal and neonatal data abstracted. Data analysis was performed from July to December 2019.

**Exposures:**

Between 16 and 21 weeks of gestation, health literacy was assessed using the Rapid Estimate of Adult Literacy in Medicine–Short Form, a validated 7-item word recognition test. In accordance with standard scoring, results were dichotomized as inadequate vs adequate health literacy.

**Main Outcomes and Measures:**

On the basis of theoretical causal pathways between health literacy and health outcomes, a priori maternal and neonatal outcomes (determined via medical records) were selected for this analysis. Multivariable Poisson regression models were constructed to estimate the associations between health literacy and outcomes. Sensitivity analyses in which education was removed from models and that excluded individuals who spoke English as a second language were performed.

**Results:**

Of 9341 participants who completed the Rapid Estimate of Adult Literacy in Medicine–Short Form, the mean (SD) age was 27.0 (5.6) years, and 2540 (27.4%) had publicly funded prenatal care. Overall, 1638 participants (17.5%) had scores indicative of inadequate health literacy. Participants with inadequate health literacy were more likely to be younger (mean [SD] age, 22.9 [5.0] vs 27.9 [5.3] years), have less educational attainment (some college education or greater, 1149 participants [73.9%] vs 5279 participants [94.5%]), have publicly funded insurance (1008 participants [62.2%] vs 1532 participants [20.0%]), and report they were a member of an underrepresented racial or ethnic group (non-Hispanic Black, 506 participants [30.9%] vs 780 participants [10.1%]; Hispanic, 516 participants [31.5%] vs 948 participants [12.3%]) compared with those with adequate health literacy. Participants who had inadequate health literacy had greater risk of cesarean delivery (adjusted risk ratio [aRR], 1.11; 95% CI, 1.01-1.23) and major perineal laceration (aRR, 1.44; 95% CI, 1.03-2.01). The adjusted risks of small-for-gestational-age status (aRR, 1.34; 95% CI, 1.14-1.58), low birth weight (aRR, 1.33; 95% CI, 1.07-1.65), and 5-minute Apgar score less than 4 (aRR, 2.78; 95% CI, 1.16-6.65) were greater for neonates born to participants with inadequate health literacy. Sensitivity analyses confirmed these findings.

**Conclusions and Relevance:**

These findings suggest that inadequate maternal health literacy is associated with a variety of adverse maternal and neonatal outcomes.

## Key Points

**Question:**

Is maternal health literacy associated with maternal and neonatal health outcomes?

**Findings:**

In this cohort study of 10 038 nulliparous individuals, those with inadequate health literacy had greater odds of cesarean delivery, major perineal lacerations, and neonates with small for gestational age status, low birth weight, and low 5-minute Apgar score, although absolute differences were generally small.

**Meaning:**

These findings suggest that inadequate health literacy is common among pregnant individuals in the US and is associated with a variety of adverse maternal and neonatal outcomes.

## Teaser

This cohort study examines the association between maternal health literacy and maternal and neonatal outcomes among nulliparous individuals in the US.

## Introduction

Health literacy is the degree to which individuals can obtain, process, and understand the basic health information and services they need to make appropriate health decisions.^1,2^ Health literacy is considered an important social determinant of health, but it is not the same as education or language.^1,2,3^ It has been well documented that even individuals with strong educational attainment and linguistic skills may have trouble obtaining, understanding, and using health information, given the complexity of the health care system and of health information. Nearly one-half of individuals in the US have low or marginal health literacy, commonly referred to as inadequate health literacy.^1,2,4^

Health literacy has been previously associated with health outcomes. There are 3 primary mechanisms through which health literacy is thought to affect health: health care access and use, patient-practitioner interactions, and health behavior, each of which may be influenced by patient and health system factors.^5,6^ These theoretical causal pathways have been demonstrated in many contexts, with inadequate health literacy linked to a variety of nonobstetric adverse health care processes, behaviors, and outcomes.^6,7^ Such adverse effects include lower use of preventive care, less adherence to treatment, more hospital stays, and higher mortality rates.^7^ Because of the deep and broad impact of health literacy on health, addressing health literacy is a national priority; objectives of the Healthy People 2020 initiative include improving health literacy as one domain of social determinants of health interventions.^8^

Despite ample data on health literacy in nonobstetric medicine, it has rarely been studied in obstetrics, and when it has been studied, it has been primarily to assess knowledge.^9^ For example, inadequate health literacy has been associated with poorer understanding of reproductive health topics, such as contraception, prenatal genetic screening, diabetes management during pregnancy, and preeclampsia.^9,10,11,12,13,14,15^ It has also been associated with disease-specific health behaviors, such as not seeking preconception care among individuals with diabetes.^9,16,17^ On the basis of the abundance of evidence on health literacy as a social determinant of health in other areas of medicine, it has been hypothesized that inadequate health literacy may deepen existing inequities in maternal and neonatal outcomes.^2,3^ However, to our knowledge, the association of health literacy with outcomes has not been studied in a general obstetric population. Thus, our objective was to evaluate the association between health literacy and maternal and neonatal outcomes.

## Methods

### Study Overview

This is a secondary analysis of data from the Nulliparous Pregnancy Outcomes Study: Monitoring Mothers-To-Be (nuMoM2b), which was a large, multicenter, observational cohort study conducted at 8 US medical centers from 2010 to 2013.^18^ Each site’s local governing institutional review board approved the study. All participants provided written informed consent before participation. The Strengthening the Reporting of Observational Studies in Epidemiology (STROBE) reporting guidelines were followed.

In this study, more than 10 000 nulliparous individuals with singleton gestations were recruited in the first trimester to undergo longitudinal assessments. Recruitment was conducted at geographically diverse locations and was designed to sample a population reflective of the general US population. Individuals were eligible for enrollment if they had a live singleton gestation, had no previous pregnancy beyond 20 weeks’ gestation, and were between 6 weeks 0 days and 13 weeks 6 days of gestation at recruitment. Exclusion criteria included maternal age younger than 13 years, history of 3 or more spontaneous abortions, current pregnancy complicated by suspected fatal fetal malformation or known aneuploidy, assisted reproduction with a donor oocyte, multifetal reduction, or plan to terminate the pregnancy.

### Data Collection

Data were collected via multiple sources, including in-person interviews, surveys, and medical record review. Participants completed 3 study visits with trained research personnel. At least 30 days after delivery, trained and certified record abstractors reviewed the medical records of all participants and their neonates and recorded final birth outcomes.^18^ Full details of the study protocol previously have been published.^18^

This analysis addresses maternal health literacy as the exposure of interest. To measure health literacy, all participants completed the Rapid Estimate of Adult Literacy in Medicine–Short Form (REALM-SF) at visit 2, which was between 16 and 21 weeks’ gestation.^18,19^ The REALM-SF is a 7-item word recognition test—the words are *behavior*, *exercise*, *menopause*, *rectal*, *antibiotics*, *anemia*, and *jaundice*—commonly used for quick assessment of health literacy. It was derived from the 66-item version of the REALM, both of which have been validated in diverse settings outside of pregnancy.^19^ Participants are shown a written list of words and asked to say each word aloud. If pronunciation is incorrect or not attempted, a point is not awarded for that word. The number of correctly pronounced words is tallied to generate the total score. Scores are categorized according to grade level using standardized scoring criteria.^19^ In nuMoM2B, the REALM-SF was performed only among participants who chose to complete interviews in English, including individuals who may have spoken English as a second language. Individuals who spoke English as a second language were included in the primary analysis because they chose to complete all aspects of the study in English, which suggests they are likely to attempt health care communication in English. This analysis included all participants with available REALM-SF data.

### Outcomes

On the basis of the established causal pathway models for health literacy,^5,6^ we hypothesized that health literacy would be associated with outcomes via their impact on pregnancy-related knowledge, behaviors, communication, and care access, all of which could be affected by patient and health system factors. Using this pathway, we selected maternal and neonatal outcomes that could plausibly be affected by factors such as patient-practitioner interactions, health behaviors, or health care use. For maternal complications, outcomes included gestational diabetes, hypertensive disorders of pregnancy, chorioamnionitis, cesarean delivery, major perineal laceration (third or fourth degree perineal laceration), postpartum hemorrhage with blood transfusion, and postpartum readmission within 30 days of discharge. Gestational diabetes diagnosis was based on clinical record review using each site’s local protocol for diagnosis; analysis of gestational diabetes excluded individuals with pregestational diabetes. Hypertensive disorders of pregnancy included antepartum, intrapartum, or postpartum (up to 14 days) gestational hypertension, preeclampsia, eclampsia, or superimposed preeclampsia, as defined by the American College of Obstetricians and Gynecologists.^20^ Perineal laceration was evaluated because of its potential association with patient-practitioner interactions. Neonatal outcomes included small for gestational age (SGA) status neonate (<10th percentile by Alexander criteria^21^), low birth weight (<2500 g), very low birth weight (<1500 g), macrosomia (>4500 g), preterm birth (<37 weeks’ gestation), admission to the neonatal intensive care unit, 5-minute Apgar score less than 4, and receipt of neonatal respiratory support. Maternal demographic and clinical characteristics measured via assessments at visit 1 (between 6 and 14 weeks) were assessed as potentially confounding factors. Self-reported race and ethnicity were assessed because they were considered important social determinants of health.

### Statistical Analysis

For this analysis, consistent with definitions and terminology of prior literature, REALM-SF scores were analyzed by dichotomizing them as inadequate vs adequate, in which inadequate scores were everything less than a high school level (ie, score <7).^19,22^ Such scores were labeled inadequate because scores do not indicate an absolute characteristic about an individual, but rather indicate a relational difference regarding the ability to have the best success in interfacing with the health care system. We examined differences between baseline demographic and clinical characteristics by health literacy status using χ^2^ and *t* tests. We assessed differences between maternal and neonatal outcomes by health literacy using χ^2^ tests. Bivariable and multivariable Poisson regression models were constructed to estimate relative risks of each outcome, with adequate health literacy as the referent. Multivariable models accounted for potential confounders determined on the basis of a priori hypotheses using a directed acyclic graph (eFigure in the Supplement). Two sensitivity analyses were performed. In the first, we excluded education from the multivariable models, given the possible collinearity of health literacy with educational attainment; all other variables from the primary model were retained in these models. Second, we excluded participants who spoke English as a second language; these models included all variables in the primary model. Self-reported race and ethnicity were not examined as effect modifiers because there is no reason the effect of health literacy should differ by racial or ethnic identity.

Analyses were performed using StataMP statistical software version 15.0 (StataCorp). All statistical tests were 2-tailed and considered significant at the *P* < .05 level. This analysis was completed from July to December 2019.

## Results

Of 10 038 participants in nuMoM2B, 9341 had REALM-SF data available for analysis and were eligible for inclusion; the mean (SD) age was 27.0 (5.6) years, and 2540 (27.4%) had publicly funded prenatal care. In this population, 1638 participants (17.5%) had inadequate health literacy. Among individuals with inadequate health literacy, most had scores at the seventh to eight grade reading level (1256 participants [76.1%]), followed by 218 participants (13.3%) at the fourth to sixth grade reading level, and 174 participants (10.6%) at the third grade level or below. Although health literacy scores were associated with education, inadequate health literacy was not limited to individuals with less educational attainment. Specifically, 57.0% of participants with an eighth grade education or less had inadequate health literacy (406 participants), as did 39.0% of those with some high school (411 participants), 23.0% of high school graduates (419 participants), 14.0% with some college education (134 participants), 7.0% with an associate’s or technical degree (185 participants), and 4.0% of college graduates (83 participants).

Participants with inadequate health literacy differed from those with adequate health literacy (Table 1). Those with inadequate health literacy were younger (mean [SD] age, 22.9 [5.0] vs 27.9 [5.3] years), more likely to self-identify as non-Hispanic Black (506 participants [30.9%] vs 780 participants [10.1%]) or Hispanic (516 participants [31.5%] vs 948 participants [12.3%]), and more likely to have public insurance (1008 participants [62.2%] vs 1532 participants [20.0%]) than those with adequate health literacy. They were less likely to be married (411 participants [25.1%] vs 5251 participants [68.2%]) or have at least some college education (1149 participants [73.9%] vs 5279 participants [94.5%]) than those with adequate health literacy. Participants with inadequate health literacy were also more likely to have obesity (492 participants [30.0%] vs 1691 participants [22.0%]) but were less likely to have used tobacco (648 participants [39.6%] vs 3275 participants [42.5%]) or have a mental health disorder diagnosed before pregnancy (235 participants [15.0%] vs 1404 participants [18.7%]) than those with adequate health literacy.

**Table 1.** Demographic and Clinical Characteristics Associated With Maternal Health Literacy

Characteristic	Health literacy, participants, No. (%)		*P* value
	Inadequate (n = 1638)^a^	Adequate (n = 7703)	
Maternal age, mean (SD), y	22.9 (5.0)	27.9 (5.3)	.004
Race and ethnicity			
Non-Hispanic			<.001
White	456 (27.8)	5265 (68.4)	
Black	506 (30.9)	780 (10.1)	
Hispanic	516 (31.5)	948 (12.3)	
Asian or other^b^	160 (9.8)	710 (9.2)	
Public insurance	1008 (62.2)	1532 (20.0)	<.001
Household income <200% of federal poverty level	637 (66.8)	1646 (24.5)	<.001
Married	411 (25.1)	5251 (68.2)	<.001
English as primary language^c^	1514 (92.4)	7692 (99.9)	<.001
Some college education or greater	1149 (73.9)	5279 (94.5)	<.001
Obesity^d^	492 (30.0)	1691 (22.0)	<.001
Ever used tobacco	648 (39.6)	3275 (42.5)	.03
Chronic hypertension	40 (2.6)	188 (2.5)	.92
Pregestational diabetes	32 (2.0)	112 (1.5)	.12
Asthma	228 (15.8)	975 (13.9)	.06
Mental health disorder^e^	235 (15.0)	1404 (18.7)	<.001

^a^ Inadequate health literacy was defined as less than high school level of health literacy based on the Rapid Estimate of Adult Literacy in Medicine–Short Form.

^b^ Other includes American Indian/Native American and any other race not listed above.

^c^ Participants included in this analysis all chose to complete the Rapid Estimate of Adult Literacy in Medicine–Short Form in English.

^d^ Obesity is defined as body mass index greater than or equal to 30 (weight in kilograms divided by height in meters squared).

^e^ Refers to mental health disorders diagnosed before pregnancy.

Maternal and neonatal outcomes by health literacy status are shown in Table 2. Hypertensive disorders of pregnancy were more frequent among participants with inadequate health literacy, but findings did not achieve statistical significance (adjusted risk ratio [aRR], 1.07; 95% CI, 0.95-1.19) after adjustment for potential confounders. Chorioamnionitis was similarly more frequent among participants with inadequate health literacy, but did not differ on multivariable analyses (aRR, 1.24; 95% CI, 0.99-1.55). In contrast, participants with inadequate health literacy had small but increased adjusted relative risks of cesarean delivery (aRR, 1.11; 95% CI, 1.01-1.23) and major perineal laceration (aRR, 1.44; 95% CI, 1.03-2.01). Risk of gestational diabetes, postpartum hemorrhage, and postpartum readmission did not differ by health literacy status.

**Table 2.** Maternal and Neonatal Outcomes by Adequacy of Health Literacy

Outcomes	Health literacy, participants, No. (%)		RR (95% CI)	
	Inadequate (n = 1638)^a^	Adequate (n = 7703)	Unadjusted	Adjusted^b^
Maternal				
Gestational diabetes	62 (4.0)	343 (4.6)	0.86 (0.66-1.13)	0.94 (0.69-1.27)
Hypertensive disorder of pregnancy	392 (25.0)	1718 (22.9)	1.09 (0.99-1.20)	1.07 (0.95-1.19)
Chorioamnionitis	128 (8.1)	474 (6.3)	1.29 (1.07-1.55)	1.24 (0.99-1.55)
Cesarean delivery	442 (28.1)	2055 (27.4)	1.03 (0.94-1.12)	1.11 (1.01-1.23)
Major perineal laceration (n = 5089)	53 (6.9)	323 (7.5)	0.93 (0.70-1.23)	1.44 (1.03-2.01)
Postpartum hemorrhage with transfusion	13 (0.8)	71 (0.9)	0.86 (0.48-1.55)	0.68 (0.35-1.31)
Postpartum readmission	31 (2.0)	123 (1.6)	1.20 (0.81-1.77)	1.15 (0.74-1.76)
Neonatal				
Small for gestational age (<10th percentile)	247 (15.8)	713 (9.6)	1.65 (1.44-1.89)	1.34 (1.14-1.58)
Low birth weight (<2500 g)	153 (9.3)	452 (5.9)	1.59 (1.34-1.90)	1.33 (1.07-1.65)
Very low birth weight (<1500 g)	34 (2.1)	87 (1.1)	1.84 (1.24-2.72)	1.36 (0.87-2.12)
Macrosomia (>4500 g)	87 (5.3)	313 (4.1)	1.31 (1.04-1.65)	1.33 (0.77-2.29)
Preterm birth (<37 wk)	153 (10.0)	623 (8.1)	1.23 (1.04-1.45)	1.02 (0.84-1.23)
Neonatal intensive care unit admission	297 (19.1)	1261 (17.0)	1.13 (1.00-1.26)	1.06 (0.93-1.22)
5-min Apgar score <4	11 (0.7)	23 (0.3)	2.29 (1.12-4.68)	2.78 (1.16-6.65)
Receipt of respiratory support	123 (35.1)	515 (32.0)	1.10 (0.94-1.29)	0.97 (0.80-1.16)

Abbreviation: RR, risk ratio.

^a^ Inadequate health literacy was defined as less than high school level of health literacy based on the Rapid Estimate of Adult Literacy in Medicine–Short Form. Referent is adequate health literacy (Rapid Estimate of Adult Literacy in Medicine–Short Form scores indicating high school education level of health literacy or greater).

^b^ Multivariable models are adjusted for maternal age, body mass index greater than 30 (weight in kilograms divided by height in meters squared), race/ethnicity, insurance, educational attainment, marital status, any mental health disorder, and tobacco history.

Regarding neonatal outcomes (Table 2), participants with inadequate health literacy had a greater frequency of SGA status neonates (247 neonates [15.8%] vs 713 neonates [9.6%]), with a 1.34-fold greater (95% CI, 1.14-1.58; *P* < .001) adjusted risk of SGA status. Similarly, participants with inadequate health literacy had greater adjusted risk of low birth weight (aRR, 1.33; 95% CI, 1.07-1.65). Very low birth weight, macrosomia, and preterm birth were more frequent among participants with inadequate health literacy but did not differ after adjusting for potential confounders. However, participants with inadequate health literacy had neonates with greater relative risk of 5-minute Apgar score less than 4 (aRR, 2.78; 95% CI, 1.16-6.65).

Both sensitivity analyses supported the findings of the primary analysis (Table 3). In both sensitivity analyses, the findings did not change, with all RRs remaining within 10% of the primary analysis.

**Table 3.** Sensitivity Analyses Examining the Association of Health Literacy With Maternal and Neonatal Outcomes

Outcomes	aRR (95% CI)	
	Sensitivity analysis 1: education removed from model^a^	Sensitivity analysis 2: excluded individuals who spoke English as a second language^b^
Maternal		
Gestational diabetes	0.99 (0.73-1.32)	0.96 (0.70-1.31)
Hypertensive disorder of pregnancy	1.05 (0.95-1.17)	1.09 (0.98-1.22)
Chorioamnionitis	1.20 (0.98-1.48)	1.29 (1.03-1.61)
Cesarean delivery	1.12 (1.02-1.23)	1.10 (0.99-1.22)
Major perineal laceration	1.40 (1.03-1.90)	1.39 (0.98-1.97)
Postpartum hemorrhage with transfusion	0.76 (0.39-1.47)	0.60 (0.30-1.21)
Postpartum readmission	1.09 (0.72-1.66)	1.20 (0.78-1.87)
Neonatal		
Small for gestational age (<10th percentile)	1.36 (1.16-1.58)	1.38 (1.17-1.62)
Low birth weight (<2500 g)	1.39 (1.13-1.71)	1.33 (1.07-1.65)
Very low birth weight (<1500 g)	1.46 (0.93-2.28)	1.30 (0.82-2.07)
Macrosomia (>4500 g)	1.21 (0.73-2.01)	1.22 (0.67-2.21)
Preterm birth (<37 wk)	1.08 (0.90-1.30)	0.99 (0.81-1.21)
Neonatal intensive care unit admission	1.09 (0.95-1.23)	1.06 (0.92-1.22)
5-min Apgar score <4	3.01 (1.26-7.21)	2.86 (1.20-6.80)
Receipt of respiratory support	0.98 (0.82-1.16)	0.93 (0.76-1.12)

Abbreviation: aRR, adjusted risk ratio.

^a^ Multivariable models are adjusted for maternal age, body mass index greater than 30 (weight in kilograms divided by height in meters squared), self-reported race/ethnicity, insurance, marital status, any mental health disorder, and tobacco history.

^b^ Analysis excluded 133 participants. Multivariable models are adjusted for maternal age, body mass index greater than 30, self-reported race/ethnicity, insurance, educational attainment, marital status, any mental health disorder, and tobacco history.

## Discussion

In this study, nearly 1 in 5 pregnant individuals had inadequate health literacy. Inadequate health literacy was associated with differences in maternal and neonatal outcomes, including greater risk of cesarean delivery, major perineal laceration, SGA status, low birth weight, and low 5-minute Apgar score. Although the absolute differences were small, these differences may have a meaningful public health impact on a population level. In addition, as has been demonstrated repeatedly in other contexts, health literacy, education, and language are discrete constructs. Health literacy is not simply a reflection of educational attainment or primary language, but rather is a distinct social determinant of perinatal health.

### Results in Context

Health literacy is associated with health learning, which has documented downstream effects on health knowledge, behaviors, and outcomes.^6^ For these reasons, health literacy has been associated with many adverse health behaviors and outcomes, including poorer use of preventive health care, suboptimal management of chronic diseases, poorer self-reported health status, increased frequency of hospitalizations, greater likelihood of medication errors, excess adult mortality, and higher health care costs.^1,7,8,23,24^ The outcomes of our study suggest that corollaries to these adverse health outcomes exist in the obstetric context. Prior limited work in obstetrics suggests that inadequate health literacy is associated with poorer pregnancy-specific knowledge and some health behaviors.^9^ Our study extends this work by demonstrating that in a population of pregnant individuals enrolled in a longitudinal study, these associations between inadequate health literacy and adverse outcomes exist. The association of health literacy with health behaviors and outcomes may hold even greater import in pregnancy than in nonobstetric settings because pregnancy is a time of rapid knowledge acquisition, increased demand for health behavior changes, and intensive need for physician counseling. Failure to achieve optimal health in pregnancy may alter the risk profile for the individual and family across the life cycle.

Health literacy influences and is influenced by multiple sociodemographic characteristics as well as other determinants across the life course, such as patient activation, social support, and culture.^6,24,25,26,27^ Effective identification of health literacy as a social determinant of health requires placing health literacy within a life course framework.^24^ For example, our findings are consistent with existing data suggesting that health literacy is not synonymous with educational attainment at the time of the assessment. In addition, our findings are from individuals experiencing one particular life stage, and literature suggests that the adequacy of health literacy changes according to life events, cognitive capacity and stressors, and medical needs.^6,24,28^ As with other complex social determinants, such as racial identity and socioeconomic status, health literacy is likely a risk marker that works in concert with other risk markers and may represent a separate but intersectional pathway through which health promotion interventions could be developed.

### Clinical Implications

Health care practitioners who care for pregnant individuals should appreciate the high prevalence of inadequate health literacy. In particular, obstetricians should take steps to communicate in an understandable manner with all patients, take into account each individual’s unique circumstances and abilities for health information comprehension, and develop and use tools and methods that are accessible to all individuals, including those with inadequate health literacy.^2^ Health literacy should be considered as a universal precaution in which all individuals are cared for with a low-health-literacy approach.^4,8,29^ Multiple actionable steps may help practitioners treat health literacy as a universal precaution.^1,2,29,30^ Strategies include adopting the approach that all patients may have low health literacy, asking open-ended questions and checking for understanding, using easy-to-read written communication, limiting the number of messages delivered in an interaction to no more than 4, using simple language and focusing on action, and supplementing written material with pictures and diagrams.^2^ Evidence from other areas of medicine suggests that interventions using these strategies can improve health-related knowledge, appropriate use of health care, adherence to medical treatment, and some health outcomes.^30,31,32,33^

### Research Implications

Future investigation must explore the mechanisms between health literacy and obstetric outcomes. Proposed mechanisms are diverse and may include differential adherence to prenatal care or health behavior guidelines (eg, nutrition and weight gain), differential understanding of counseling, alterations of the effects of psychosocial stressors, or differential engagement or self-advocacy.^9,34^ In addition, inadequate health literacy may compound practitioner biases or exacerbate poor patient-practitioner communication. Better understanding of pregnancy-specific mechanisms may support the development of evidence-based interventions to improve health literacy. Recent data on health literacy interventions to improve pregnancy outcomes indicate that such work remains nascent and largely focused on improving knowledge.^35^ Next steps must address the potential impact of interventions to improve health literacy and whether such interventions improve health, as well as greater study on the unique learning and motivation features of pregnancy.^36^ The role of health literacy in postpartum morbidity, including perinatal depression, also warrants investigation.^9,15^

In addition, health literacy is complex and multifaceted.^28^ Future investigations should investigate other aspects of health literacy, such as print literacy, oral literacy, health numeracy, and comprehension, in obstetrics.^23^ Studying the full meaning of health literacy also includes understanding how different types of health literacy may be more relevant for certain populations. For example, health numeracy skills^37,38^ may be particularly important for individuals with gestational diabetes, who must manipulate numbers to self-manage nutrient intake and medication administration.^12^

### Strengths and Limitations

A major strength of this study is the use of a large sample of pregnant individuals that is representative of the US population. The cohort is extraordinarily well characterized and includes detailed prospective assessments of health literacy prior to the occurrence of outcomes.^18^ The assessment of health literacy via the REALM-SF substantially enhances the fidelity of literacy evaluation, in contrast to investigations that use education as a proxy for literacy.

However, there are limitations to consider. As with all studies of health literacy, this study is observational and subject to unmeasured confounding. Second, health literacy is a skill that may evolve, and this study captured health literacy at a single time point in midpregnancy; however, it is unlikely that health literacy levels would be expected to change substantively during pregnancy. Third, as noted already, the REALM-SF has limitations as a measure of health literacy. For example, it is primarily an assessment of reading and pronunciation, rather than comprehension, and, in contrast to newer measures, it does not comprehensively assess all types of health literacy, such as health numeracy. Fourth, although this is one of the largest studies of health literacy in pregnancy, the study may be underpowered to detect differences in uncommon events, such as postpartum readmission. Fifth, participants were enrolled in a longitudinal investigation that began in early pregnancy and were recruited from large medical centers; thus, the findings may not be fully generalizable. The young age of the population and their enrollment in this study may be underlying reasons for the lower prevalence of inadequate health literacy than that of general population.

## Conclusions

In summary, this cohort study found that inadequate health literacy was common among pregnant individuals in the US. Health literacy is an important, yet often underrecognized, social determinant of health, and may be associated with health disparities. Our work suggests that health literacy may be independently associated with the risk of adverse perinatal outcomes, separate from educational attainment and language. These findings represent a call to action for the development of evidence-based interventions to optimize communication and enhance health education.

## Notes

Supplement. **eFigure.** Directed Acyclic Graph
