# Supplementary material for: Association of Health Literacy Among Nulliparous Individuals and Maternal and Neonatal Outcomes
Source: JAMA Netw Open. 2021 Sep 1;4(9):e2122576. doi: 10.1001/jamanetworkopen.2021.22576 (PMC8411280; doi:10.1001/jamanetworkopen.2021.22576)
Supplement: Supplement. — eFigure. Directed Acyclic Graph [file jamanetwopen-e2122576-s001.pdf]

Supplemental Online Content

Yee LM, Silver R, Haas DM, et al. Association of health literacy among nulliparous individuals and maternal and neonatal outcomes. *JAMA Netw Open*. 2021;4(9):e2122576. doi:10.1001/jamanetworkopen.2021.22576

eFigure. Directed Acyclic Graph

This supplemental material has been provided by the authors to give readers additional information about their work.

© 2021 Yee LM et al. *JAMA Network Open.*

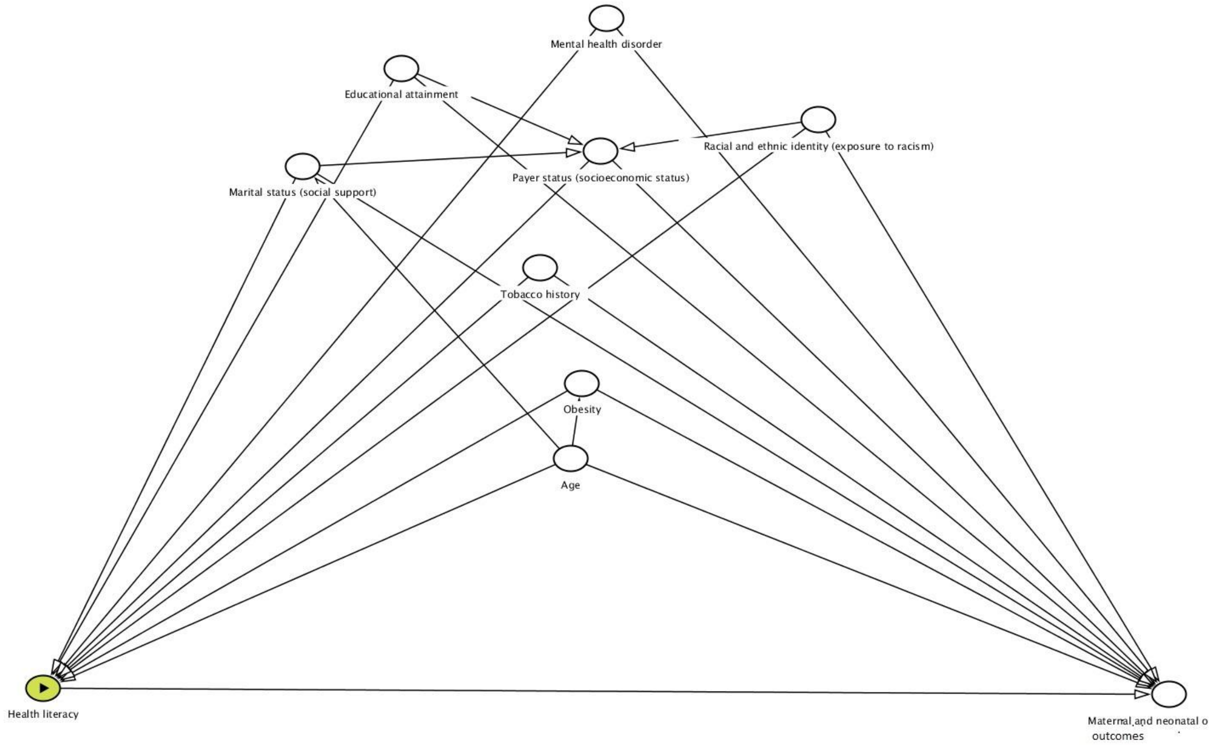

eFigure. Directed Acyclic Graph

© 2021 Yee LM et al. *JAMA Network Open.*
